# Techno-Functional Properties and Applications of Inulin in Food Systems

**DOI:** 10.3390/gels11100829

**Published:** 2025-10-15

**Authors:** Elisa Canazza, Miriam Grauso, Dasha Mihaylova, Anna Lante

**Affiliations:** 1 Department of Agronomy, Food, Natural Resources, Animals, and Environment—DAFNAE, University of Padova, Viale dell’Università, 16, 35020 Legnaro, Italy; elisa.canazza.3@phd.unipd.it (E.C.); miriam.grauso@studenti.unipd.it (M.G.); 2 Department of Microbiology and Biotechnology, University of Food Technologies, 26 Maritza Blvd., 4002 Plovdiv, Bulgaria; dashamihaylova@yahoo.com

**Keywords:** inulin, inulin-based gels, gelation mechanisms, fat replacer, texture modification, dietary fiber, plant-based products, sustainable food design

## Abstract

Inulin, a type of fructan primarily extracted from chicory, Jerusalem artichoke, and dahlia, is a prebiotic dietary fiber increasingly valued for its multifunctional roles in food systems. Beyond its well-established nutritional benefits linked to gut microbiota modulation and metabolic health, inulin also provides unique techno-functional properties that make it a versatile structuring ingredient. This review emphasizes inulin’s ability to form gel-like networks and emulsion gels, examining the mechanisms of gelation and the influence of chain length, degree of polymerization, and processing conditions on gel stability and performance. Inulin-based gels act as effective fat replacers, texture modifiers, and carriers of bioactive compounds, supporting the reformulation of foods with reduced fat and sugar while maintaining desirable texture and sensory quality. Applications span a wide range of food systems, including dairy, meat, bakery, confectionery, plant-based, and gluten-free products, where inulin contributes to enhanced structure, stability, and palatability. Furthermore, the potential to obtain inulin from agro-industrial by-products strengthens its role in sustainable food design within a circular economy framework. By integrating nutritional, structural, and technological functionalities, inulin and inulin-based gels emerge as promising tools for the development of innovative and health-oriented food products.

## 1. Introduction

In today’s food innovation landscape, reformulating products to meet health, sensory, and sustainability demands presents both challenges and opportunities. Functional ingredients like dietary fibers that can deliver structural benefits while supporting nutritional goals are at the forefront of this transformation [1]. Inulin, a naturally occurring Fructan-Type polysaccharide predominantly extracted from chicory roots (*Cichorium intybus*), tubers of Jerusalem artichoke (*Helianthus tuberosus*), and dahlia (*Dahlia pinnata*) [2], has gained increasing attention for its dual role as bioactive dietary fiber and versatile techno-functional ingredient in food systems [3]. In the nutrition and food science literature, including this review, the term ‘inulin’ is often used broadly to encompass all inulin-type fructans that selectively nourish beneficial intestinal microbiota, thus promoting host health [2,4,5,6,7,8,9,10]. Structurally, they consist of fructose units linked by β-(2→1) glycosidic bonds, which resist hydrolysis by human digestive enzymes in the upper gastrointestinal tract [11,12]. In the colon, they act as selective substrates for beneficial bacteria, particularly *Bifidobacterium* and *Lactobacillus* species, which possess β-fructofuranosidase enzymes (EC 3.2.1.26). Fermentation by these microorganisms leads to the production of short-chain fatty acids (SCFAs) as primary end products. These metabolites exert various physiological effects on the host, underpinning many of the health benefits attributed to prebiotics [5,13]. Once absorbed, these metabolites contribute minimally to caloric intake, providing approximately 6.3 kJ/g (1.5 kcal/g) [2,14].

From a technological standpoint, inulin is highly valued for its ability to form particle gels consisting of crystalline micro-domains dispersed in water, creating textures that mimic fat [15,16,17]. This unique quality makes it invaluable in reduced-fat formulations across various food categories, including dairy, meat, bakery, confectionery, snacks, gluten free and plant-based products [15,18,19,20,21,22,23,24,25]. Beyond fat replacement, inulin also modifies texture, increases bulk with minimal caloric load, and acts as a stabilizer in dispersed systems. By increasing the viscosity of the continuous phase, reducing droplet mobility, and forming a protective layer around dispersed particles, inulin enhances the physical stability of emulsions and foams [18,26,27,28]. These properties are particularly advantageous in emulsion gels, where inulin not only improves stability and texture but also serves as an effective carrier matrix for bioactive compounds. This enables their encapsulation, protection, and controlled release [26,27,29,30,31,32]. These same capabilities are increasingly applied in nutraceutical and pharmaceutical contexts. Researchers have investigated inulin-based gels as carriers for the targeted delivery of bioactive molecules, probiotics, and therapeutic agents. These applications benefit from inulin’s biocompatibility, biodegradability, and prebiotic activity [26,33,34,35,36,37,38,39]. In this broader framework, inulin stands out as a strategically relevant ingredient, combining multifunctional properties that enable the development of health-oriented products while fulfilling structural and sensory quality requirements. Its plant-based origin, compatibility with clean-label formulations [26,40,41], and potential sourcing from agro-industrial by-products [42,43,44,45,46] align with circular economy strategies, reinforcing its value in sustainable food design.

This review provides a comprehensive analysis of inulin’s techno-functional properties in food systems, emphasizing its gelation behavior, structuring capacity, and role in texture modulation. Structure–function relationships are discussed alongside applications across diverse food sectors, highlighting strategies to reformulate healthier products without compromising sensory attributes. Additionally, the physiological benefits of inulin are considered in relation to its technological functions, underscoring how formulation variables and processing conditions influence gel structure, stability, and performance. By integrating nutritional, technological, and sustainability perspectives, this review positions inulin and inulin-based gels as multifunctional tools for developing next-generation foods and highlights future opportunities for their broader application in innovative food design.

## 2. Chemical Structure of Inulin

**Figure 1:**
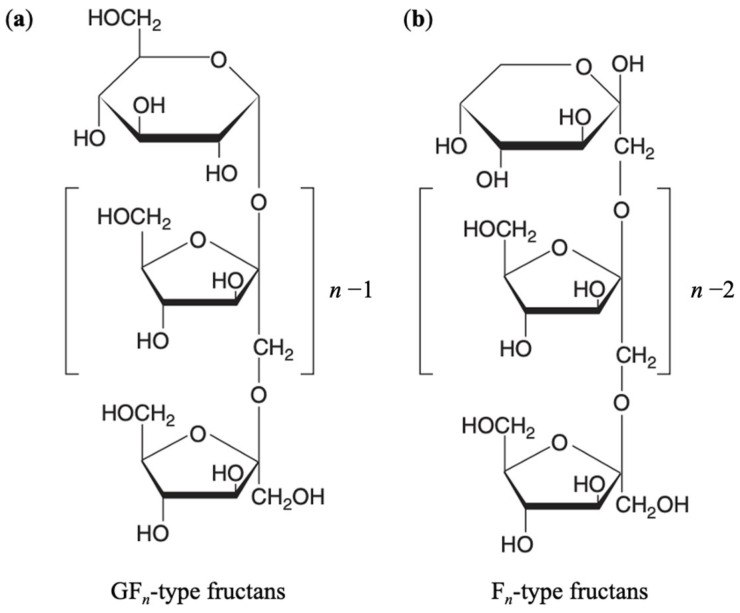
Chemical structure of inulin: (**a**) GF*_n_*-type fructans, composed of β-(2→1)-linked fructose units terminated with a glucose residue via an α-(1→2) linkage; (**b**) F*_n_*-type fructans lacking the terminal glucose residue.

Chemically, inulin consists of 2 to 60 fructose units linked by β-(2→1) glycosidic bonds. The terminal fructose unit is often linked to a glucose residue by an α-(1→2) bond, as in sucrose [47]. This carbohydrate polymer is generally described by the formula GF*_n_*, where G represents the glucose terminal unit, F is the fructose residue, and *n* denotes the number of fructose units [48]. Inulin molecules are predominantly linear, though a low degree of branching (approximately 1%) can occur through β-(2→6) linkages [28,49,50]. Due to the β-configuration at the anomeric C2 of fructose, inulin-type fructans resist hydrolysis by human digestive enzymes, classifying them as non-digestible carbohydrates [3,51]. Structurally, inulin contains both amorphous domains, rich in hydroxyl groups engaged in hydrogen bonding, and crystalline regions stabilized by intermolecular hydrogen bonds forming crystallites [51]. Inulin terminating with a glucose residue (GF*_n_* type fructans) lacks an aldehyde or ketone functional group, and therefore has no reactive or reducing end, which makes it fairly stable [52]. In contrast, inulin lacking the terminal glucose unit (F*_n_* type fructans), also known as inulo-*n*-oses with β-(2→1) linkages, possesses reducing properties and exhibits lower stability [52,53]. Figure 1 illustrates the chemical structure of inulin, including both GF*_n_* and F*_n_* type fructans.

The average molecular weight (MW) and Degree of Polymerization (DP) of inulin vary depending on the botanical source, harvest time, and extraction or post-extraction processes [47,54]. Based on DP, it can be classified into three groups: short-chain inulin (DP ≤ 10), native inulin (DP 2–60), and long-chain inulin (DP ≥ 23) fractions [55]. The short-chain forms (DP ≤ 10), also referred to as fructooligosaccharides (FOS) [56,57], are typically obtained in the food industry by partial enzymatic hydrolysis of long-chain inulin using endoinulinase (EC 3.2.1.7). In contrast, long-chain fractions are generally recovered through physical separation techniques [23]. As previously reported, inulin is sometimes used as a generic term for the entire inulin-type fructans category, in most contexts it predominantly denotes fractions with DP > 10, thereby distinguishing them from FOS [9,10].

The rheological and thermal properties of inulin are significantly influenced by temperature, concentration, and chain length. Short-chain inulin exhibits high solubility, good water-holding capacity, and mild sweetness (approximately 30–35% that of sucrose). These characteristics make it suitable as a bulking agent, partial sugar replacer, and fiber-enriching ingredient [5,16,58]. In contrast, long-chain inulin is characterized by low solubility, yet high viscosity and strong gel-forming capacity, which underpin its functionality as a fat replacer, gelling agent, thickener, and stabilizer across various food systems [16,59,60,61]. The DP and branching ultimately determine inulin’s functional attributes, including melting point, gelation capacity, glass transition temperature, and gel stability. Consequently, fractionation by DP can yield ingredients with tailored physicochemical and techno-functional properties, allowing for more precise applications in food science and technology [2].

## 3. Sources of Inulin in Plants

Inulin is found in more than 36,000 plant species belonging to both monocotyledonous and dicotyledonous groups [62]. Dietary intake of inulin varies considerably according to geographical region and eating habits. In Western diets, reported values range from 1 to 10 g/day, with lower intakes observed in the United States (≈1.3–3.5 g/day) compared to European populations (≈3–11 g/day) [52]. The main botanical families rich in inulin include Compositae, Liliaceae, Asparagaceae, Gramineae, and Amaryllidaceae, where they are predominantly stored in roots, tubers, and bulbs [4]. Table 1 summarizes the occurrence of inulin in different plant sources, specifying the tissues involved and the inulin content expressed as grams per hundred grams of Fresh Weight (FW).

As reported above, several commonly consumed plant-based foods contribute amounts of inulin to the human diet [2,51,62,63]. The highest concentrations occur in Jerusalem artichoke and dahlia tubers, and in chicory and yacon roots. Among these, chicory, Jerusalem artichoke, and dahlia stand out as the main raw materials for industrial inulin production due to their high content. Dahlia tubers yield about 25 t/ha (15–22% Dry Matter, DM) and 2.5–3 t/ha of inulin (DP 13–20), while Jerusalem artichoke produces 35–60 t/ha (19–25% DM) and 4.5–8.5 t/ha of inulin (DP 6–10). Chicory roots, the current dominant source, achieve 25–75 t/ha (20–25% DM) with 5–11 t/ha of inulin (DP 10–14). Despite its richness, dahlia is mainly cultivated for ornamental purposes, and Jerusalem artichoke faces limitations related to tuber morphology, soil adherence, and sensitivity to low temperatures. Chicory, by contrast, combines high yields, regular roots, and strong cold tolerance, making it the most suitable and widely used crop for large-scale inulin extraction in temperate regions [2,8,64,65].

Concurrently, increasing attention has been devoted to the recovery of inulin from agro-industrial by-products and wastes, especially those from globe artichoke processing. This industry generates significant biomass (about 80–85% of FW), primarily consisting of bracts, stalks, and leaves. While these parts are unsuitable for direct human consumption, they show potential as raw materials for recovering high-value ingredient aligning with the circular economy principles [2,8,64,65].

Globe artichoke synthesizes inulin with chain lengths of up to 200 units, and high MW fractions isolated from processing residues—primarily external bracts—show an average DP of 46, higher than typically reported for Jerusalem artichoke, chicory, and dahlia [2,66]. Several studies have explored this valorization pathway: Garcia-Castelló et al. (2022) achieved ~80% inulin recovery from artichoke residues through Hot-Water Extraction (HWE), along with substantial phenolic content and antioxidant activity [67]. Zeaiter et al. (2019) reported the recovery of long-chain inulin (DP 32–42) from bracts and stems using ultrasound-assisted extraction followed by ethanol precipitation, yielding fractions containing over 70% inulin [68]. Soto-Maldonado et al. (2020) [69] demonstrated that sequential extraction of artichoke canning discards with hot water and hydroalcoholic solvents provided up to 10.9 g inulin/100 g dry waste, along with significant amounts of phenolics. Beyond artichoke, other studies have explored inulin extraction from various sources; Lara-Fiallos et al. (2021) [70] successfully optimized aqueous extraction from garlic processing waste, obtaining ~8 g inulin/100 g dry artichoke discards along with significant phenolic content, Lopes et al. (2017) [71] isolated both inulin and FOS from *Stevia rebaudiana* stems, an underutilized by-product of the sweetener industry.

## 4. Extraction and Isolation of Inulin

### 4.1. Conventional Inulin Production Process

The conventional inulin production process typically involves three main steps: extraction, purification, and drying [8,72]. Initially, inulin-rich raw materials are thoroughly cleaned, dried, and mechanically processed (e.g., sliced or pulped) to maximize surface area for efficient extraction [73]. The extraction phase primarily utilized Hot Water Extraction (HWE), where plant material is infused in water at 70–80 °C for 1–2 h, solubilizing hydrophilic components, including inulin [8,65]. Most industrial methods rely on HWE, with minor variations in extraction time and temperature. Agitation or continuous stirring is often employed to enhance mass transfer and extraction efficiency [2]. While robust and widely adopted, conventional HWE is energy and water intensive. The elevated temperatures promote the co-extraction of impurities, necessitating additional clarification steps. Moreover, intensive liming and carbonation can introduce residual calcium ions, requiring removal during downstream polishing. These constraints have driven the development of more sustainable green extraction technologies aimed at improving yields, reducing resource consumption, and preserving polymer integrity [3,51,65,74,75]. Following extraction, the raw juice undergoes multistage purification to eliminate co-extracted impurities such as peptides, degraded proteins, colloidal particles, and inorganic anions [8,76].

**Figure 2:**
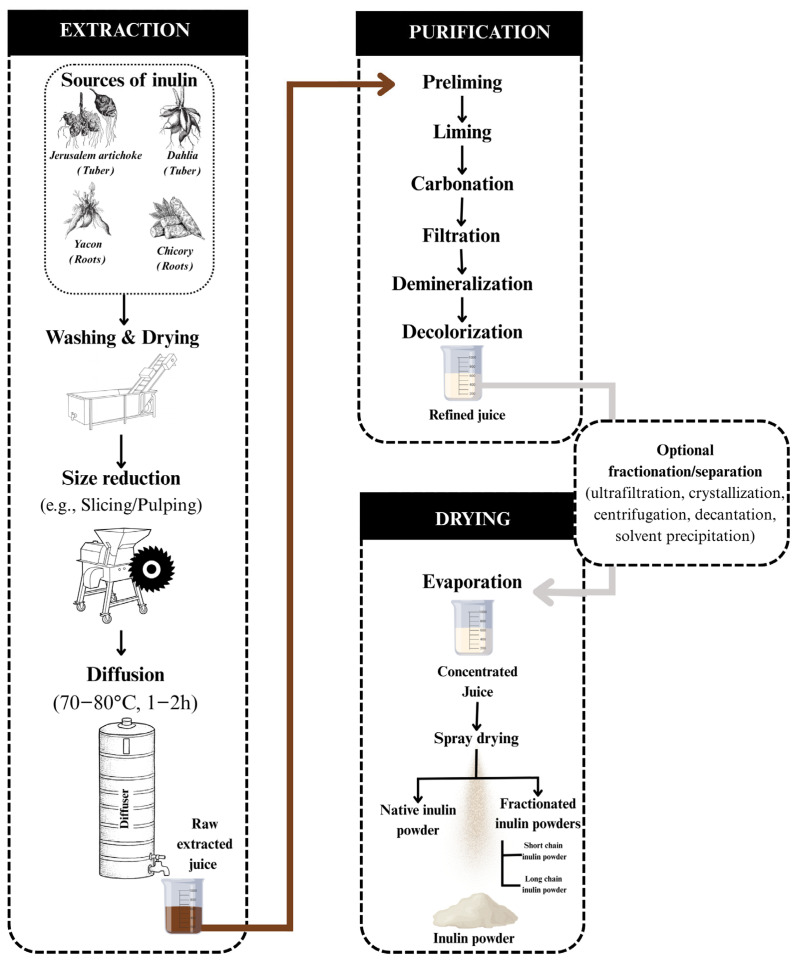
Schematic representation of conventional inulin extraction and purification process from various plant sources [3,65,72].

The purification process typically begins with pre-liming, liming, and carbonation, where precipitation on CaCO_3_ sludge is followed by filtration. To reduce protein contaminants, enzymes or protein-removal reagents—such as lime milk–phosphoric acid, Sevag reagent, or trichloroacetic acid—may be employed [26,77,78]. The pre-purified juice then undergoes refinement using cationic and anionic ion-exchange resins for demineralization. Decolorization is achieved with either activated carbon or macroporous resins. While activated carbon is effective, it often causes product losses. In contrast, macroporous resins offer high adsorption capacity with minimal inulin loss [3,8]. Depending on the targeted DP and MW distribution, complementary operations may include ultrafiltration, crystallization, centrifugation, or decantation [8]. Long-chain inulin fractions can be selectively precipitated from aqueous solution by adding high concentrations of organic solvents such as methanol, ethanol, propanol, acetonitrile, or acetone. Although acetone and acetonitrile generally achieve more efficient precipitation, ethanol is preferred in food applications due to its GRAS status and relatively low boiling point (78.3 °C), facilitating solvent recovery and recycling [8,65,74,79,80]. Post-purification, the clarified inulin solution is typically sterilized by microfiltration (usually 0.2 μm pore size) [65]. The concentrated juice, which may optionally undergo fractionation, is then dried to a residual moisture content of approximately 5% [3,65]. For producing stable, microbiologically safe, and commercially viable inulin powders, spray drying is currently the most practical and widely applied method. While freeze-drying yields slightly higher quality products, it is substantially more costly [2,8,65]. Figure 2 presents a schematic overview of this conventional process.

Key process variables such as temperature, extraction time, pH, and solid-to-liquid ratio significantly impact recovery yield and polymer stability [3,7,8]. These parameters require optimization based on the specific plant material and processing conditions [12].

Inulin’s solubility significantly increases with temperature. However, extended exposure to high temperatures, especially in acidic conditions (pH ≤ 4), leads to hydrolysis. This process reduces the DP and triggers Maillard-derived browning, which in turn complicates downstream purification processes [47,76,81].

Conventional inulin extraction typically involves immersing plant material in hot water, followed by purification with CaCO_3_, ion-exchange resins, and activated carbon. However, this process has faced criticism due to its high energy consumption and the necessity for multiple purification steps [75]. In response to these challenges, researchers have developed alternative methods that address these limitations. These new approaches aim to reduce energy usage, enhance environmental sustainability, improve scalability, shorten processing times, and increase both yield and purity. The growing demand for inulin has heightened the importance of selecting efficient and sustainable extraction strategies. As a result, various non-conventional techniques have emerged as promising alternatives for inulin extraction. In the following sections, we will discuss the main innovative methods that have been applied to this process, highlighting their advantages and potential for industrial application [65,75,77,82].

### 4.2. Non-Conventional Extraction Techniques of Inulin

#### 4.2.1. Ultrasound-Assisted Extraction (UAE)

Ultrasound refers to sound waves with frequencies above the human hearing range (20 kHz–1 MHz), widely applied in food processing. When transmitted through liquids or liquid–solid systems, ultrasound generates alternating compression and rarefaction cycles, leading to cavitation. The growth and collapse of cavitation bubbles disrupt cell walls, enhance mass transfer, and facilitate solvent penetration into plant tissues. These effects accelerate the release of intracellular compounds and make UAE a highly attractive non-conventional extraction technique [65,83,84]. Compared with conventional HWE, UAE generally operates at lower temperatures, requires shorter extraction times, and achieves higher yields and purity with reduced energy consumption. Its main limitations concern the need for specific reactor design to avoid “blind spots,” where ultrasound waves fail to propagate effectively [65]. The extraction mechanism involves mainly two physical phenomena: (i) the diffusion across the cell walls and (ii) the cell content rinsing after breaking the walls [85]. Owing to these advantages, UAE has become one of the most extensively studied methods for inulin recovery, consistently showing superior performance to conventional HWE across different plant matrices.

Lingyun et al. (2007) optimized UAE from Jerusalem artichoke tubers (neutral pH, 20 min, 77 °C, 11:1 solvent-to-solid ratio), obtaining an 83.6% yield [86]. UAE reached maximum yield in only 8 min, compared with 16 min for HWE, and indirect sonication was more suitable than direct, which caused partial degradation [86]. Milani et al. (2011) studied burdock roots and showed that UAE under optimal conditions (25 min, 83% amplitude, 37 °C) yielded about 24% inulin, more than double that obtained by HWE from the same material (11%) [87]. Singh et al. (2025) optimized UAE of chicory roots (120 min, 60 °C, 1:40 g/mL), achieving 64.79% yield with >95% purity, outperforming conventional extraction (59.1% yield at 90 °C for 6 h), while also enhancing antioxidant activity (53.8% vs. 45.2% for HWE) [88].

#### 4.2.2. Microwave-Assisted Extraction (MAE)

Microwaves are electromagnetic fields with frequencies ranging from 300 MHz to 300 GHz and wavelengths between 1 and 1000 mm [65,89]. Their heating effect is based on the interaction with polar molecules, where electromagnetic energy is converted into thermal energy through ionic conduction and dipole rotation. In extraction processes, MAE promotes rapid and uniform heating, generating high internal pressure within plant tissues, which enhances cell wall disruption and facilitates solvent penetration. The extraction mechanism using MAE involves three sequential steps: (i) the separation of solutes from the active sites of the sample matrix under increased temperature and pressure; (ii) the solvent’s diffusion across the sample matrix; and (iii) the release of solutes from the sample matrix to the solvent [65]. In recent years, MAE has gained growing attention for the extraction of bioactive components and polysaccharides [81]. Compared with conventional methods, MAE offers several advantages, including faster heating, shorter extraction times, reduced thermal gradients, smaller equipment size, and higher yields [90,91]. Due to its several benefits, MAE was successfully applied for inulin extraction. Moreover, MAE can sterilize the extract and inactivate oxidases [82]. Due to these benefits, MAE has also been successfully applied for inulin extraction, showing strong potential as a scalable and sustainable alternative to traditional HWE. Demirci et al. (2023) optimized inulin extraction from Jerusalem artichoke by comparing HWE and MAE [92]. Under optimized conditions, MAE achieved yields up to 91.8%, outperforming HWE (85.6%). Following ultrafiltration, the purity of inulin increased to 94–95%. In addition to yield and purity, MAE significantly enhanced functional properties: solubility was higher (94.7% vs. 91.5% for HWE), wettability and dispersibility improved, and viscosity increased, indicating better hydration and thickening capacity of the inulin powders obtained [92]. Similarly, Mudannayake et al. (2025) compared HWE and MAE for the recovery of inulin from roots of different *Asparagus* species, which represent by-products of asparagus cultivation and could be considered among the best alternative sources of fructans [93]. Both methods were tested at a fixed solid-to-solvent ratio (1:12 g/mL) and 80 °C, but MAE (600 W, 20 min) consistently yielded higher inulin contents. The best result was obtained with *A. gonocladus*, where MAE produced an inulin powder with 92.3% content, outperforming not only HWE but also commercial chicory inulin (73.4%). Beyond yield, MAE-extracted inulins retained strong antioxidant activity (≈92% DPPH inhibition) and contained significant amounts of phenolics and flavonoids, which were absent in commercial chicory inulin [94].

#### 4.2.3. Enzyme-Assisted Extraction (EAE)

EAE has been widely applied for cell wall disintegration, thereby facilitating the release of intracellular compounds and improving extraction efficiency. It has been well documented that adding specific enzymes (e.g., cellulase, α-amylase, and pectinase) during the extraction process, enhances the recovery of high-added value compounds [65]. Since inulin is typically bound within plant cell walls as protein–polysaccharide conjugates, the use of enzymes during extraction can significantly enhance its recovery [82]. Compared with conventional HWE, EAE offers several advantages, including mild operating conditions, reduced energy consumption, shorter processing times, high specificity, and the absence of solvent-derived impurities [65,77,82]. Santo Domingo et al. (2020) investigated EAE of pectin and inulin from *Cynara cardunculus* (artichoke) bracts and stems, which represent agro-industrial by-products [95]. Microwave-assisted heating was first used to obtain alcohol-insoluble residues, followed by enzymatic digestion with protease and hemicellulase. The isolated fractions contained 31.8–45.0 g/100 g of inulin and 22–29 g/100 g of galacturonic acid, with yields around 13–15%. Compared with fractions obtained from conventional heating, the MAE+EAE process produced higher levels of inulin and pectin, while operating with shorter extraction times and lower solvent use [95]. Duda et al. (2025) investigated EAE of inulin from witloof chicory roots (*Cichorium intybus* var. *foliosum*) using pectinase, alone and in combination with pressure-assisted extraction [96]. The study showed that EAE alone yielded 43.9 g/100 g DM of inulin, while combining enzymatic and pressure treatments significantly improved recovery, reaching 50.9 g/100 g DM. Importantly, EAE enabled extraction under milder conditions, reducing thermal degradation of bioactives, and resulted in extracts with lower cytotoxicity compared to pressure-only or combined treatments, highlighting its potential as a gentle and efficient strategy for inulin recovery [96].

#### 4.2.4. Pulsed-Electric Field Assisted Extraction (PEFAE)

PEFAE represents a promising non-thermal technology for enhancing the extraction of bioactive compounds from plant matrices. The process involves applying short electrical pulses (typically 0.1–80 kV/cm, with durations in the μs–ms range) to a material placed between two electrodes. Exposure to the electric field induces electroporation, namely the formation of reversible or irreversible pores in cell membranes, which increases permeability and facilitates the release of intracellular contents [65]. According to the electromechanical model, the electric field induces a reorganization of charges across the membrane, leading to an increase in transmembrane potential. Once this potential exceeds a critical threshold, electrocompressive forces cause dielectric rupture of the membrane, resulting in pore formation. This phenomenon, generally recognized as the main mechanism of electroplasmolysis, underlies the efficiency of PEFAE in improving mass transfer and extraction yields [97]. Loginova et al. (2010) [98] demonstrated that PEFAE pretreatment (100–600 V/cm) significantly accelerated the extraction of soluble matter, including inulin, from chicory roots. Compared to conventional thermal extraction (70–80 °C, >1 h), PEF reduced the activation energy of diffusion from ~263 kJ/mol to 30–40 kJ/mol, enabling efficient extraction even at lower temperatures (20–40 °C). This confirms the potential of PEF for “cold” inulin extraction with reduced energy consumption and faster kinetics. Zhu et al. (2012) [99] evaluated inulin extraction from PEFAE-treated chicory roots under industrial conditions using a pilot-scale countercurrent extractor. Optimal parameters (0.6 kV/cm, 50 ms) enhanced diffusion efficiency, yielding ~12 g/100 mL in juice at 80 °C versus ~11.5 g/100 mL in the control, and allowed a reduction in diffusion temperature by 10–15 °C while maintaining comparable inulin levels. Notably, maximum inulin recovery in pulp was achieved at 30 °C (~30 g/100 g) compared to 15 g/100 g at 70 °C, highlighting significant energy savings and an estimated profit increase of 34.76 EUR/ton of inulin. Similarly, Rivera et al. (2024) [97] applied PEFAE to *Agave americana* and found that, although yields were slightly lower than conventional extraction, the method enabled processing at lower temperatures (60 °C vs. 80 °C) and produced inulin of superior quality, characterized by higher DP (>38 fructose units) and greater thermal stability.

#### 4.2.5. Supercritical Fluid Extraction (SFE)

SFE has been extensively applied for the recovery of high-value compounds, particularly essential oils, offering the advantage of obtaining solvent-free extracts since the supercritical fluid can be readily removed by depressurization. The supercritical state, achieved above the critical temperature and pressure, combines gas-like transport properties (diffusivity, viscosity, surface tension) with liquid-like features (density and solvation power), thereby enabling shorter extraction times and higher yields compared to conventional techniques. Among available solvents, carbon dioxide is generally preferred due to its low critical point (≈31 °C, 73 bar), safety (non-toxic, non-flammable, odorless, tasteless, and inert), and low cost, making it particularly suitable for food-related applications [65,74]. Singh et al. (2024) compared SFE with Soxhlet extraction for inulin recovery from chicory roots and, under optimized conditions (398.2 bar pressure, 68.65 °C temperature, 115.11 min extraction time, CO_2_ flow rate of 10 g/min, and ethanol co-solvent flow rate of 2 mL/min), achieved a significantly higher yield (79.39% vs. 64.43%), with purity above 97% and improved antioxidant activity [100]. However, the application of SFE to inulin extraction remains scarcely explored, and further studies are needed to evaluate its feasibility and scalability for industrial use.

Non-conventional extraction techniques of inulin offer clear advantages in terms of extraction efficiency, preservation of inulin structure, and reduced environmental impact. However, they often require costly equipment and precise optimization of multiple parameters, which currently limits their large-scale application. As a result, conventional HWE remains the most widely employed method in industrial practice. Nonetheless, the growing attention to sustainability, combined with continuous advances in green extraction technologies, suggests that non-conventional methods will play an increasingly important role in the industrial landscape [44,65,101,102].

## 5. Physicochemical Properties and Gelation Behavior of Inulin

### 5.1. Solubility

The solubility of inulin is primarily influenced by its MW, DP, and temperature [12]. Short-chain inulin (low MW, DP ≤ 10) dissolves easily in water at room temperature, while solubility decreases significantly as chain length increases. Native inulin typically has a solubility of about 6% at 10 °C, which increases to 33–35% at 90 °C. This increase reflects the disruption of crystalline domains and intermolecular hydrogen bonding at higher temperatures [3,12,82]. Inulin’s solubility also varies depending on the solvent. It is sparingly soluble or insoluble in alcohols such as methanol, ethanol and isopropanol, a property utilized for precipitation and fractionation. In contrast, inulin is highly soluble in dimethyl sulfoxide [3,6,12]. Structural factors play a crucial role in solubility as well. Commercial long-chain crystalline fractions of chicory inulin (e.g., Raftiline^®^ HP, ST) have poor solubility and tend to form turbid solutions [82]. Conversely, enzymatically synthesized inulin with an intermediate DP (16–18) and lacking very long chains (DP > 30) exhibits comparatively higher solubility. This underscores the importance of MW distribution and crystallinity in determining solubility, as average DP values alone do not fully explain solubility behavior [26].

The crystalline structure of inulin significantly influences its solubility. Early research identified four polymorphic forms or isoforms (α, β, γ, δ), each with distinct MW distributions and dissolution behaviors [103,104]. The γ form is consists solely of high-MW chains (>8000 g/mol), while α and β forms also include lower-MW fractions. Notably, β inulin dissolves readily at room temperature (≈23 °C), whereas the other forms require heating to dissolve. These polymorphs exhibit different levels of thermodynamic stability, increasing in the order β < α < γ < δ. More recent studies have expanded the classification, identifying seven crystalline polymorphs in addition to amorphous forms. The solubility of these forms has been ranked as follows: α − 1 > α − 2 > γ > δ > ζ > ε > ω [105,106].

This expanded understanding of inulin’s polymorphic forms provides valuable insights into its diverse solubility characteristics, which are crucial for various applications in food science and technology.

Solubility and crystalline polymorphs play a crucial role in the nucleation and crystallization processes that drive gel formation. Inulin’s solubility level significantly impacts gel rigidity during cooling. Concurrently, the presence of undissolved inulin crystal seeds is essential for nucleation and the development of the gel network, as further discussed in Section 5.4.1. As the solution cools, molecules with higher DP tend to recrystallize first, forming nuclei due to their lower solubility and thermodynamic fluctuations. Subsequently, shorter chains progressively deposit onto the outer regions of the growing crystallites, contributing to the gel structure [107,108].

### 5.2. Sweetness

The sweetness of inulin is strongly correlated with their DP. Short-chain inulin fractions known as FOS, are highly soluble and provide a mild sweetness, approximately 30–35% that of sucrose, making them effective sugar substitutes in reduced-calorie foods [16,109,110,111]. These fractions also act as bulking agents, enhancing mouthfeel and improving the sensory balance of formulations when combined with high-intensity sweeteners like aspartame and acesulfame [51,64,112]. In contrast, long-chain inulin, characterized by low solubility and minimal sweetness, primarily contributes to gelling and texturizing properties rather than flavor enhancement [112]. From a nutritional standpoint, inulin provides approximately 1.5 kcal/g while exerting prebiotic effects, making them particularly suitable for formulations targeting weight management and glycemic control [4,5,12,73,113].

### 5.3. Physicochemical Stability

Inulin demonstrates high thermal stability, remaining largely intact up to 100 °C under neutral pH [106]. However, its β-(2→1) linkages are vulnerable to acid-catalyzed hydrolysis, particularly at pH ≤ 4. This hydrolysis is exacerbated by elevated temperatures, extended heating times, and higher moisture content, resulting in the release of fructose and glucose [26,64,82,114]. Hydrolysis negatively impacts inulin’s functionality by reducing its DP, altering MW distribution, and diminishing gelling and textural properties [3,26,47]. Additionally, inulin’s stability is critically affected by moisture content due to its marked hygroscopicity. While water uptake can delay dehydration in food systems and prolong shelf life, absorbed water acts as a plasticizer, lowering the glass transition temperature (*T_g_*) [82,115]. *T_g_* is a crucial physicochemical parameter that significantly impacts the technological performance of inulin in industrial processes. It represents the temperature at which amorphous materials shift from a rigid glassy state to a more flexible rubbery phase, resulting in a marked increase in molecular mobility [116]. Inulin typically exhibits *T_g_* ranges of 103 to 158 °C, which varies depending on factors such as chain polymerization, water activity, and the molecular structure. Notably, the *T_g_* of inulin decreases as its MW decreases [26]. When the temperature exceeds *T_g_*, the amorphous matrix becomes more viscous, leading to several undesirable effects such as stickiness, particle agglomeration, caking, and reduced encapsulation efficiency. Conversely, higher *T_g_* values offer advantages in industrial applications, particularly in enhancing the storage stability of inulin-based powders by minimizing moisture absorption and recrystallization [117,118,119].

Typically, high MW inulin absorbs less moisture at high water activity, reflecting the contribution of water-insoluble polymeric fractions. Depending on hydration level, inulin can exist in three states: amorphous phase (≤13.6 g/100 g water) with *T_g_*~150 °C, rubbery phase (14.8–15.7 g/100 g), and crystalline state (>16 g/100 g), where no glass transition is observed. Conversely, in low-moisture matrices, reduced water availability limits hydrolysis, conferring higher chemical and thermal stability [3,106,108,116]. Crystalline polymorphs of inulin display distinct thermodynamic stabilities, generally following the order β < α < γ < δ [103].

### 5.4. Gelation Mechanisms and Rheological Behavior

#### 5.4.1. The Inulin Particle–Gel Model

**Figure 3:**
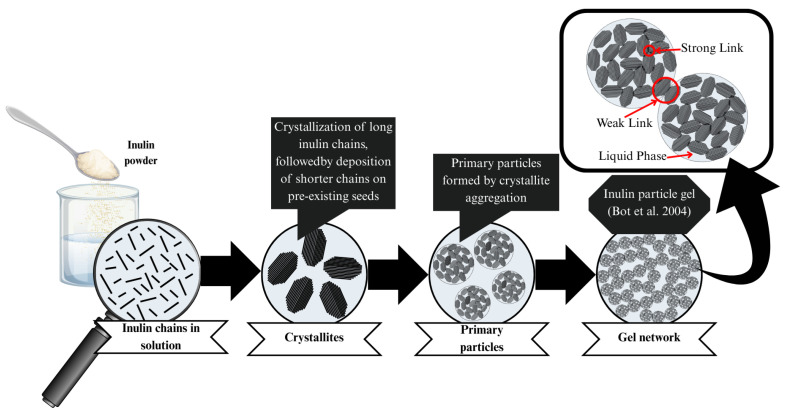
Hierarchical inulin particle–gel model. This schematic illustrates the stepwise process of inulin gel formation: dissolved inulin chains crystallize into crystallites, which then aggregate to form primary particles. These primary particles subsequently assemble into a complex gel network (Redrawn and adapted from Bot et al. (2004) [120] and Joshi et al. (2018) [121]).

The gelation process of inulin is commonly described by the particle–gel model proposed by Bot et al. (2004) [120], which conceptualizes gelation as a hierarchical, multistep assembly pathway [121]. When inulin is added to water, partial dissolution occurs, dependent on concentration and temperature [121]. As the solution cools, selective recrystallization begins, influenced by chain length. The long-chain fractions, due to their lower solubility, initiate nucleation forming crystalline seeds that serve as scaffolds for further growth. Pre-existing crystalline fragments or undissolved material can also act as nucleation sites, reducing the energetic barrier for crystallization and accelerating crystal growth [107,121]. As crystallization progresses, shorter inulin chains deposit onto these seeds, enlarging and reinforcing them. This process results in the formation of non-spherical crystallites, approximately 100 nm diameter, which constitute the fundamental structural units of the inulin particle–gel [29,107,120,121]. Continued crystallization leads to the association of these crystallites into compact aggregates, stabilized by strong intermolecular interactions arising from crystalline surface contacts and crystallization energy. These aggregates form the primary particles of the particle–gel network [11,29,121,122]. Subsequently, these primary particles assemble into irregular clusters (1–5 μm) with a fractal nature [47,121]. The aggregation of these clusters is primarily driven by van der Waals forces, leading to the development of a loosely connected yet extensive network. This hierarchical aggregation process culminates in the formation of a continuous three-dimensional network, whose pores and meshes effectively trap water. The immobilization of water within this network is responsible for the viscoelastic and gel-like properties characteristic of inulin systems [107,121,123]. The average DP of inulin significantly influences the structure of the particle–gel network, affecting packing density and gel particle size. These structural characteristics, in turn, determine the physical properties of the final gels [120,121]. This model underscores the analogy between fat and inulin particle gels, where primary particles assemble into random networks exhibiting fractal features at specific length scales [121]. A schematic representation of this hierarchical particle–gel model is illustrated in Figure 3.

The final mechanical properties of inulin gels are significantly influenced by the size, density, and connectivity of their crystalline domains. Gels consisting of numerous small, densely packed crystallites exhibit greater firmness and resistance to syneresis. In contrast, networks dominated by fewer, larger aggregates tend to be softer and more pliable. Rapid nucleation promotes the formation of numerous small crystallites, resulting in strong, highly crosslinked gels. Conversely, slower nucleation under extensive solubilization generates fewer seed crystals, leading to weaker, looser network structures [47,107,120].

#### 5.4.2. Key Processing Parameters Affecting Inulin Gelation

Temperature and concentration play crucial roles in modulating the kinetics and efficiency of inulin gelation. At low dissolution temperatures (≈25 °C), incomplete solubilization results in abundant undissolved material that serves as nucleation sites. This generates a dense network of small crystallites, yielding gels with firm textures. At moderate temperatures (40–70 °C), partial solubilization preserves crystallizable high-DP fractions alongside undissolved seeds, promoting efficient nucleation and homogeneous network formation [107,114,121,124]. Under these conditions, the volumetric gel index (VGI) approaches 100% at concentrations as low as15% *w*/*v* [123]. Gels formed within this temperature range typically exhibit smoother and more homogeneous textures. This is because heating enhances chain mobility, facilitating controlled nucleation during the cooling [123,125]. For example, Han et al. (2021) [126] produced gels by heating to 70 °C for 5 min followed by 5 h of cooling. These gels demonstrated the storage modulus G′ > G″ across various frequencies, shear-thinning behavior, and adjustable elastic modulus. Notably, these properties have been associated with extended colonic residence time and positive modulation of gut microbiota in vivo [126]. Above 80 °C, gel strength decreases significantly. At 15% *w*/*v* the VGI drops to approximately 75% at 80 °C and to about 15% at 90 °C. This decrease occurs primarily because high temperatures enhance solubilization and melt crystallites, reducing the number and size of nuclei available to maintain the network [123]. Under neutral conditions, chemical hydrolysis of the β-(2→1) linkages is limited. However, at pH ≤ 4, these bonds undergo acid-catalyzed hydrolysis. The hydrolysis rate constant (*k*) increases with temperature according to the Arrhenius equation [47], resulting in shorter chains, reduced crystallizability, and a higher critical gelation concentration (from ~15% at 40–60 °C to ~40% at 100 °C). At such extreme conditions, network formation is severely compromised or absent, leading to weak or non-gelling systems [47,120,127]. Mechanical forces also play a crucial role. Shear during processing, ranging from mild stirring to high-pressure homogenization, alters particle–particle contacts and can reduce the critical concentration for gelation [57,120,127]. When mixing for 5 min at 25 °C, complete gelation (VGI = 100%) requires ≥30% *w*/*v* under low shear (~250 rpm), whereas under high shear (~5000 rpm) a percolated network forms already at ~15% *w*/*v* [47]. Concentration is pivotal in governing gelation. Below 10%, inulin merely increases viscosity without network formation [123]. Between 10 and 30%, viscosity rises sharply, and incipient gel-like structures appear. Above 30%, the storage modulus (G′) rapidly increases, reflecting the transition to elastic-dominant behavior and the establishment of a percolated crystalline network. In the 40–50% range, gelation becomes nearly instantaneous, producing continuous, homogeneous networks. Beyond 50%, gels evolve into firm, rigid matrices that nevertheless preserve fat-like sensory properties [3,47,106,123]. Rheologically, this transition is reflected in the sharp rise in G′, which increases from weakly structured values of 10^2^–10^3^ Pa near the gelation threshold (25–27.5%) to about 10^5^ Pa at ~40% [128].

In addition, other factors significantly modulate inulin gelation. Within the pH range 4–9, gelation remains generally unaffected. However, under strongly acidic conditions, hydrolysis dominates and prevents network formation [47,81]. In acidic environments, hydrolysis may be restricted to less than 10% when the products contain high DM (>70%) or are kept at refrigeration temperatures (<10 °C) [11]. Solvent polarity also plays a crucial role in network assembly. Co-solvents such as ethanol or glycerol accelerate gelation by reducing solvent–polymer interactions and enhancing inulin–inulin associations. This leads to faster crystallization kinetics and shorter induction times [123]. The cooling rate significantly affects nucleation. Rapid cooling favors the formation of numerous small crystallites, resulting in compact, firm gels. Conversely, slow cooling promotes fewer, larger crystals, producing softer textures [120,123].

Interactions with other biopolymers, such as proteins or polysaccharides, can markedly affect gelation. In some systems, these macromolecules compete for water, limiting inulin solubilization and delaying crystallization [63,129,130,131]. In others, they act as structuring partners, reinforcing the network through hydrogen bonding or electrostatic interactions. For example, incorporating plant proteins (pea, soy) or polysaccharides (sodium alginate, chitosan) has been shown to produce more compact and cohesive gels, significantly improving firmness, yield stress, and spreadability [47,129,132,133,134,135].

#### 5.4.3. Role of Processing Methodologies

Besides the factors discussed above, the method of gel induction significantly influences structure and texture. Thermal induction, involving heating followed by cooling, is the most common approach. It drives a sol–gel transition through crystallization upon cooling [123,125,136]. While simple and widely used, this method has limitations when processing thermolabile components. Moreover, excessive solubilization at high temperatures may remove undissolved inulin crystallites that act as nucleation sites, potentially impairing crystallization and gel formation, ultimately weakening the network [120,123,127,137]. Mechanical shear can promote gelation at lower concentrations by disrupting aggregates and enhancing particle–particle interactions, even without full solubilization [47,57,120,123,127]. The choice of processing device also affects gel strength: dispersions processed with a colloid mill yield the weakest gels, rotor–stator mixers (e.g., Ultra-Turrax^®^) produce intermediate firmness, while high-pressure homogenizers generate the strongest and most compact structures [11,114,119,123,138].

Emerging non-thermal technologies such as ultrasound and High Hydrostatic Pressure (HHP) show promise in modulating nucleation, crystallization, and particle–particle interactions, thereby improving homogeneity, stability, and tunability of inulin gels [137,139]. Ultrasound-assisted gelation is a sustainable, additive-free strategy that alters hydrogen bonding and crystallinity without modifying the primary molecular structure. Spectroscopic and structural analyses (FTIR, XRD, particle size) have confirmed that ultrasound treatment enhances consistency, hardness, and adhesiveness of long-chain inulin gels, yielding finer microstructures. The most pronounced effects were observed at high acoustic intensity (~1735 W/cm^2^ for 10 min), where particle rearrangements favored denser and more stable networks [140]. HHP, typically up to 1000 MPa, promotes gelation by reorganizing non-covalent interactions while preserving covalent linkages. This pressure-driven compaction limits molecular mobility, producing hydrogels with improved firmness, viscoelasticity, and water retention compared to thermally induced counterparts [130,131,136,137,139].

Chemical crosslinking offers another approach to gel formation. Strategies such as methacrylation with free-radical polymerization, UV or electron beam irradiation, and esterification reactions yield covalently stabilized gels with enhanced mechanical strength and responsiveness. However, these methods are more complex and raise concerns regarding toxicity and biocompatibility, requiring further optimization [136].

## 6. Technological Applications of Inulin in Food Systems

### 6.1. As Fat Replacer and Texture Modifier

Reducing dietary fat in food formulations is a major challenge in modern food technology. Lipids are crucial for sensory and technological quality, contributing to lubrication, creaminess, tenderness, aeration, and flavor release [2,16,141]. Their removal often results in poor mouthfeel, altered texture, reduced stability, and lower consumer acceptance [141,142]. However, excessive fat intake is linked to obesity, hypercholesterolemia, cardiovascular disease, type 2 diabetes, and hypertension [143,144]. To address these issues, fat replacers have been developed to reproduce the functional and sensory roles of fat while lowering caloric value. These are generally classified as fat substitutes (chemically modified lipids used gram-per-gram), fat mimetics (mainly carbohydrate- or protein-based ingredients reproducing rheological and sensory roles), and fat analogs (fat-like molecules with reduced digestibility) [142].

Long-chain inulin has gained considerable attention as a carbohydrate-based fat mimetic. Due to its low solubility, it forms particle gels composed of submicron microcrystals that aggregate into clusters, entrapping water and reproducing the rheological and sensory functions of lipids [29,121]. This network organization closely resembles the crystalline structure of fats, explaining its ability to provide creaminess, spreadability, and mouthfeel [29,121]. Additionally, inulin offers health benefits associated with its dietary fiber and prebiotic nature, making it particularly valuable for low- and zero-fat formulations [2,29,121]. Long-chain inulin shows enhanced fat-mimetic capacity, compared to native inulin, generating smooth and creamy textures without detectable particles and interacting with proteins to further strengthen the matrix [23,57,145,146]. Inulin gels are characterized by low yield strain and fracture strain, resulting in short, soft, and easily spreadable textures that closely mimic semisolid fats such as margarines [2,76,120]. In food systems, inulin acts as a fat replacer primarily in water-continuous matrices, where the particle–gel network stabilizes the aqueous phase and enhances creaminess. In emulsified systems, it localizes in the aqueous phase, where it increases viscosity and contributes to stabilization.

**Figure 4:**
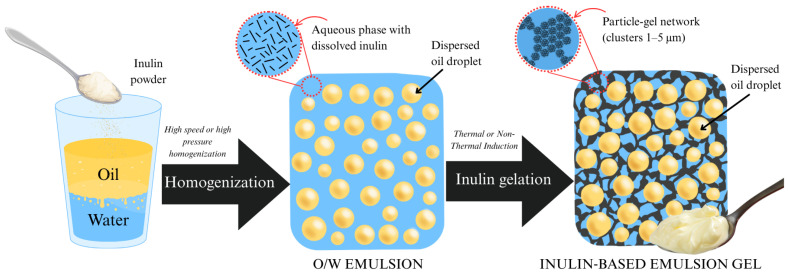
Schematic illustration of inulin-based emulsion gel formation. The process involves the dispersing of oil droplets into a pre-gel inulin solution, followed by gelation to create a stable, fat-mimicking structure.

Beyond hydrogels, inulin can also participate in emulsion gels, where oil droplets (dispersed phase) are immobilized within a continuous gel network. Emulsion gels can be prepared either by embedding an emulsion into a pre-formed gel phase or by dispersing oil into a pre-gel polymer solution that subsequently undergoes gelation [147,148]. A schematic representation of the latter approach is shown in Figure 4.

These systems enhance rheological properties, storage stability, and provide fat-like textures [11,27,149]. A significant advantage is their ability to reduce total fat content while structuring lipid fractions enriched in mono- and polyunsaturated fatty acids. This results in products with the creamy consistency of conventional fats but enhanced nutritional quality [29,150,151,152,153]. Moreover, inulin enhances the functional profile of foods by contributing dietary fiber. According to European regulations, products can be labeled as “source of fiber” or “high fiber” if they contain more than 3 g or 6 g/100 g of fiber, respectively [154]. Inulin’s capacity to replace fat—especially saturated fat—while adding fiber and prebiotic benefits has garnered widespread industry interest and proven effective across diverse food matrices.

For instance, in meat products, De Souza Paglarini et al. (2022) [155] demonstrated that inulin-based gelled emulsions could replace 50–100% of pork back fat in Bologna sausages. This reduced total fat by up to 31%, improved the fatty acid profile, and allowed for “source” or “high source of fiber” claims, without affecting significantly consumer acceptance [155]. Jayarathna et al. (2022) [24] used garlic inulin hydrogels (1–3%) to replace 3% vegetable oil in chicken sausages. This reduced fat content from 13.7% to 4.5–4.9%, with 2% addition identified as the most acceptable for flavor and overall quality. García et al. (2006) [156] evaluated the incorporation of inulin (2.5–7.5%, *w*/*w*) in conventional (23% fat) and reduced-fat (10% fat) mortadella-type sausages, comparing its addition as powder and as pre-hydrated gel. Fat reduction of approximately 44% resulted in a 33% decrease in caloric value, while inulin effectively compensated for texture and mouthfeel losses typically associated with fat removal. Instrumental texture analysis showed that powdered inulin increased product hardness, particularly in reduced-fat formulations, whereas the gel form produced softer, more cohesive textures. Sensory evaluation confirmed good overall acceptability up to 7.5% inclusion, with the gel form yielding juicier and more palatable products, simulating the lubricity and creaminess of fat without adversely affecting flavor [156]. Other strategies include protein–inulin combinations, such as that proposed by Rodríguez Furlán et al. (2014) [157], where 2% inulin combined with 2.5% bovine plasma proteins replaced 20–35% of fat in minced meat while maintaining emulsion stability and sensory properties. Totaro et al. (2025) [60] developed low- and high-DP inulin gels (≈39–52% inulin with guar gum), for use in in burgers, reducing fat content from 12% to 5%. Both gels improved cooking yield and reduced shrinkage, with high-DP gels showing superior water retention and dimensional stability [60]. These examples illustrate the versatility and effectiveness of inulin as a fat replacer in various meat products, offering both nutritional improvements and maintained sensory qualities.

In dairy products, inulin has proven equally versatile. Tiwari et al. (2015) [158] replaced up to 40% of milk fat with 2–6% inulin in ice cream, reducing fat from 9.7% to 3.8%. Formulations with 2–4% inulin maintained sensory acceptance comparable to full-fat control [158]. Samakradhamrongthai et al. (2021) [21] optimized reduced-fat ice cream with 4.02% inulin, lowering fat by ~2.3%. Gomez-Betancur et al. (2020) [159] showed that fortifying yogurt mousse with 4.8% long-chain inulin and calcium restored creaminess and viscoelasticity to levels comparable to full-fat products. Wongkaew et al. (2025) [160] improved texture and consumer acceptance of low-fat goat feta by combining inulin and polydextrose. Moghiseh et al. (2021) [161] enriched mozzarella with inulin (DP ≈ 23) and kefiran, increasing protein and moisture content. This yielded firmer and more cohesive structures, albeit with lower meltability. Kant et al. (2024) [162] highlighted the potential of combining inulin with whey proteins in paneer, achieving better texture, higher sensory scores, and longer shelf life.

In bakery and confectionery, where fat reduction often compromises tenderness and flavor, inulin plays a strategic role [16,163]. Paciulli et al. (2020) [152] used inulin–olive oil emulsion gels to replace 20–50% of butter in shortbread cookies. This significantly reduced saturated fat content, with ≥40% replacement allowing the “reduced saturated fat” claim. Reformulated cookies showed higher volume, harder texture, and darker color. They remained stable over 60 days of storage, with only minor texture changes, and no rancid off flavors were detected [152]. Rodríguez-García et al. (2012) [164] demonstrated that replacing (35–70%) of sunflower oil with inulin in sponge cakes produced softer crumbs and good aeration, although complete substitution reduced sensory quality. Zahn et al. (2010) [165] showed that 50% fat replacement in muffins preserved palatability, whereas full substitution resulted in denser, less acceptable products. In bakery products, complete substitution is more problematic than in other food matrices, since fat supports gluten network formation and air incorporation. Replacing it with inulin leads to a stiffer gluten structure and impaired gas retention, due to the “gluten-diluting effect” and water competition. As a result, loaf expansion is reduced, yielding denser crumbs and lower sensory quality, which explains why full replacement in these products remains limited [3,165,166,167,168]. In the context of fat-reduced confectionery products, Tavares Filho et al. (2025) [169] evaluated how the individual addition of inulin or xylooligosaccharides (XOS) at a total fiber content of 2.5 g/100 g affected the sensory perception of dulce de leche prepared with either whole or skimmed milk. The study showed that samples produced with whole milk were associated with more positive emotions and overall preference, whereas those made with skimmed milk generated less favorable responses. Inulin and XOS mitigated the “too greasy” sensation typical of full-fat formulations and promoted an ideal sweetness perception, evidencing a cross-modal interaction between sweetness and fat-related creaminess. Conversely, the skimmed milk formulation with added XOS was perceived as “too thick,” reflecting the higher viscosity conferred by oligosaccharides and the lack of fat-induced lubrication. Structurally, these differences are consistent with the distinct polymeric organization of the fibers: inulin forms cohesive hydrogen-bonded networks capable of retaining water and mimicking fat-like creaminess, whereas XOS, with shorter xylose chains, exhibits weaker water-binding and gel-forming capacity, leading to softer and less cohesive textures. From a flavor standpoint, the partial compensation of sweetness and mouthfeel by inulin enhanced the acceptability of low-fat samples, indicating its potential as a multifunctional ingredient acting both as a fat mimetic and sweetness modulator [169].

Inulin is gaining attention also in the rapidly growing field of plant-based formulations. Narala et al. (2022) [170] investigated pea protein-based vegan ice cream, where 2–4% inulin improved overrun, meltdown resistance, and texture. However, higher levels (6–8%) negatively affected flavor and overall acceptability, highlighting the importance of dosage optimization in these matrices [170].

These studies collectively demonstrate inulin’s effectiveness as a fat replacer across various food categories, offering both nutritional improvements and maintained sensory qualities when used at optimal levels.

### 6.2. As Sugar Replacer

Reducing added sugars remains a major challenge for the food industry. Common sugars like sucrose, glucose, and fructose serve not only as sweeteners but also as key techno-functional ingredients in food systems. Their roles extend beyond imparting sweetness: act as bulking agents, contribute to flavor and color development through thermal reactions, improve tenderness in baked products by modulating gluten–starch interactions, control crystallization in confectionery, stabilize frozen desserts by limiting ice crystal growth, influence water activity, critical for extending shelf life in products such as jams, jellies, and canned fruits [16,171,172,173]. Replacing natural sugars is complex, as alternative sweeteners must simultaneously reproduce both sensory attributes and structural functions. Artificial sweeteners such as acesulfame K, aspartame, sucralose, and saccharin are widely employed as zero-calorie alternatives. These are several hundred times sweeter than sucrose and non-cariogenic. However, their safety and long-term health effects remain debated, raising concerns over their broad application [171,174,175,176,177,178]. This situation underscores the challenge of identifying sugar replacers that provide health benefits, replicate the structural and textural functions of sucrose, match the sensory profile of sugar, ensure consumer acceptance. The ideal sugar replacer would address all these aspects while offering nutritional advantages, making the search for effective alternatives an ongoing priority in food science and technology. In this context, inulin has emerged as a promising sugar replacer, combining technological versatility with nutritional advantages. Short-chain fractions (DP < 10) provide mild sweetness (approximately 30–35% of sucrose) along with bulk, water-binding capacity, and contribution to body and mouthfeel. These properties make inulin suitable for partial or total sugar substitution across various product categories [16,64,76,110,111].

From a nutritional standpoint, inulin is not hydrolyzed in the upper gastrointestinal tract due to their β-(2→1) linkages and therefore do not raise blood glucose. Its glycemic index is extremely low (approximately 14 for native inulin, compared to 100 for glucose) [76,179], while its caloric value (1–1.5 kcal/g) is far below that of conventional sugars (4 kcal/g) [76,179,180]. These properties make inulin, particularly its short-chain forms, highly suitable for formulating reduced-glycemic foods, with specific relevance for populations with (pre)diabetes [181,182,183]. Several applications confirm these benefits. Tsatsaragkou et al. (2021) [173] investigated inulin as a sugar replacer in cakes and biscuits. They found that at 30% replacement, low-DP inulin (Orafti^®^) produced cakes with crumb structure, texture, and mouthfeel comparable to sucrose-based controls. In contrast, high-DP inulin (Fibruline^®^ Instant) increased batter viscosity and resulted in denser crumbs. In biscuits, low-DP inulin yielded softer, less crunchy products [173]. Harastani et al. (2021) [184] tested inulin and green banana flour as combined fat- and sugar-replacers (10–30%) in industrial muffins. The reformulated products achieved nutritional claims such as “reduced sugar,” “reduced fat,” and “high fiber,” with acceptable sensory properties up to 30% replacement. Inulin was key to maintaining moistness and palatability [184]. More recently, Berk et al. (2024) [185] evaluated sucrose substitution in cocoa–hazelnut spreads using an inulin–stevia blend (90:10). Up to 80% replacement reduced water activity and improved stability while preserving rheological and melting properties close to the control. However, full replacement (100%) compromised spreadability [185]. Overall, inulin—particularly short-chain inulin—offers a combination of mild sweetness, fiber enrichment, and very low glycemic impact. These properties position it as versatile sugar replacers in bakery, confectionery, spreads, and other reduced-sugar formulations.

### 6.3. As Fiber Enrichment

Inulin’s versatility extends beyond its use as a fat and sugar replacer. While it contributes to improved texture, creaminess, and stability, inulin is often incorporated specifically to enhance the fiber content of foods. This enables the use of nutritional claims such as “source of fiber” or “high in fiber” [154], combining technological functionality with health-promoting properties and reinforcing the role of inulin as a multifunctional ingredient for healthier product design [64,65].

Several studies have demonstrated these benefits in different food matrices. Cascone et al. (2023) [186] formulated functional chestnut sweet creams enriched with pumpkin pulp and inulin, partially replacing sucrose. The addition of 6% inulin doubled the fiber content, lowered water activity, and improved nutritional quality while reducing caloric density [186]. Çetin Babaoğlu et al. (2021) [187] investigated the use of inulin-rich Jerusalem Artichoke Powder (JAP) in sourdough bread. JAP addition enhanced nutritional properties by increasing total dietary fiber, phenolic compounds, and antioxidant activity, while reducing the estimated glycemic index. However, high substitution levels (>10%) negatively impacted loaf volume, crumb texture, and sensory acceptability [184]. Padalino et al. (2017) [168] investigated the fortification of whole-meal durum wheat spaghetti with 2–4% (*w*/*w*) inulin of different DP, comparing a high-DP inulin extracted from cardoon roots with a low-DP commercial chicory inulin. The addition of inulin significantly increased the total dietary fiber content and reduced starch digestibility, improving the nutritional profile of the pasta. However, low-DP inulin negatively affected texture and sensory properties, increasing adhesiveness and bulkiness and reducing firmness due to its high solubility and water-binding capacity, which weakened the gluten matrix and altered dough viscoelasticity. Conversely, high-DP inulin, characterized by lower solubility and higher MW, interacted more favorably with the protein–starch network, leading to a firmer and more cohesive structure with improved mouthfeel [168].

Inulin has also been widely applied in liquid matrices, particularly for the formulation of fiber-enriched beverages. In fruit-based systems, Cassani et al. (2017) [188] enriched strawberry juice with an inulin–FOS blend combined with ultrasound processing and vanillin addition, achieving a significant increase in dietary fiber content while maintaining acceptable sensory properties and color stability [188]. Beyond this, numerous studies have explored the incorporation of FOS and inulin as functional supplements in a variety of beverages, including apple juice, cranberry juice, carrot juice, and orange juice [189,190,191,192,193,194]. Pereira et al. (2025) [195] demonstrated that the incorporation of *Lacticaseibacillus casei* and inulin, as probiotic and prebiotic components, respectively, in peach and grape juice enhanced the nutritional profile (vitamin B12, antioxidant capacity, ACE inhibition) while maintaining probiotic stability throughout storage [195]. Collectively, these studies identified fruit juices as effective carrier systems for FOS/inulin, suitable for the development of functional beverages aimed at improving nutritional quality and promoting consumer health and well-being. However, it is important to highlight that beverage processing under high-energy conditions, especially in acidic environments (pH ≤ 4), may promote inulin hydrolysis [47,190,191,192]. Therefore, optimizing processing parameters is essential to preserve the structural integrity and prebiotic functionality of inulin in beverage formulations. Han et al. (2018) [196] compared the incorporation of various dietary fibers (inulin, polydextrose, xanthan gum, and acacia gum) in corn-based extruded snacks to develop high-fiber formulations. Among the tested fibers, inulin significantly increased the product’s bulk density and hardness, while reducing expansion and porosity. Although these structural effects slightly reduced crispness, inulin enhanced the dietary fiber content and contributed to a more compact, uniform texture, demonstrating its functionality as both a nutritional and structural modifier in fiber-enriched snack products [196].

### 6.4. Other Applications of Inulin in the Food Industry

Beyond its well-established use as a fat and sugar replacer, inulin has also been investigated in other innovative applications within the food sector. One promising area is its use as an encapsulating agent in delivery systems. Thanks to its gel-forming ability, inulin can entrap sensitive molecules, enhance their stability and enable controlled release—applications that extend to both food and pharmaceutical sectors [27,136,197,198].

Recent studies have demonstrated inulin’s potential in various encapsulation applications. Dagni et al. (2025) [199] encapsulated *Dysphania ambrosioides* essential oil and α-terpinene with inulin and arabic gum. They achieved 88% encapsulation efficiency, stability above 80%, and bio-accessibility over 40%. The resulting microcapsules preserved antibacterial activity and oxidative stability, highlighting inulin’s potential as a carrier for volatile bioactives in food applications [199].

Ahmed et al. (2024) [200] successfully encapsulated cinnamon essential oil into an inulin matrix by freeze-drying. At 15% essential oil concentration, encapsulation efficiency reached ~63%, with strong antimicrobial effects against *Salmonella enterica* serovar Typhimurium and *Listeria monocytogenes.* This supports its use in functional foods, pharmaceuticals, and even active packaging [200]. Widianto and Puangpraphant (2024) [201] showed that blends of maltodextrin and inulin (10% + 30%, respectively) achieved ≈92% encapsulation efficiency for betacyanin extract from red dragon fruit peel. This blend also improved retention of bioactives and increased bio-accessibility (57.1% vs. 35.9% in free extract). Furthermore, the encapsulation system enhanced color stability, reduced hygroscopicity, and extended shelf life, confirming the potential of inulin-based carriers for stabilizing natural pigments [201]. Wyspiańska et al. (2019) [202] evaluated the efficiency of inulin as a wall material for the microencapsulation of soy isoflavones in isotonic beverages. Inulin-based microcapsules showed a higher encapsulation efficiency (64%) and greater retention of total isoflavones during spray-drying and storage compared to maltodextrin. Morphological analysis by scanning electron microscopy revealed that capsules obtained with inulin exhibited a more homogeneous and smoother surface. From a sensory standpoint, beverages fortified with inulin-encapsulated isoflavones were rated more favorably than those containing pure extracts, as microencapsulation effectively masked the undesirable taste, odor, and color of isoflavones. During simulated gastrointestinal digestion, microencapsulated isoflavones displayed a gradual release profile, confirming the protective and controlled-release properties of inulin as a carrier matrix [202]. In addition, inulin has recently attracted interest as a natural cryoprotectant in frozen foods. Unlike synthetic cryoprotectants, which may raise safety concerns, inulin represents a safe, plant-derived alternative. Its hydrocolloid properties allow it to form hydrogen bonds with water, limiting ice nucleation and crystal growth during frozen storage. This results in better texture, reduced drip loss, and improved quality preservation [203]. Recent studies have demonstrated inulin’s effectiveness as a cryoprotectant in various food applications. Görgüç and Yılmaz (2023) [204] showed that ultrasound-assisted vacuum impregnation of inulin into sour cherries reduced drip loss by 13–18% and preserved phenolics, anthocyanins, and overall quality, particularly when combined with IQF freezing [204]. Bayraktar et al. (2024) [203] confirmed similar benefits in beef, where inulin-treated samples showed reduced thawing and cooking losses, lower ion leakage, and improved color and lipid stability during six months of frozen storage. Moreover, Cao et al. (2022) [205] highlighted how chain length influences cryoprotective action in silver carp surimi. Short-chain inulin (8%) preserved gel strength and water-holding capacity comparable to sucrose–sorbitol. Long-chain inulin (8%) proved even more effective at reducing thawing losses, in some cases surpassing conventional cryoprotectants [205].

An increasingly relevant application of inulin is in gluten-free products, where it provides both nutritional and technological functions. The absence of gluten proteins typically leads to doughs with poor elasticity, low loaf volume, and crumb fragility [49,206,207]. In gluten-free formulations, hydrocolloids such as hydroxypropyl methylcellulose, xanthan, guar gum, and psyllium husk are widely employed to enhance dough viscoelasticity, gas retention, and loaf volume [208,209,210]. Inulin generally provides complementary benefits when combined with other conventional hydrocolloids. Acting through its water-binding and gel-forming properties, it enhances dough hydration, crumb porosity, and delays staling, while simultaneously enriching gluten-free products with soluble fiber and lowering glycemic response [166,206,207,211,212].

Burešová et al. (2024) [213] reported that inulin addition up to 30% in rice-based dough enhanced bread volume, crumb porosity, and softness while reducing hardness and chewiness. However, excessive substitution (≥40%) compromised product quality. At such high concentrations, inulin competes with starch for available water, delaying gelatinization and reducing dough viscosity. This imbalance weakens gas retention, leading to a denser, less cohesive crumb, and lower sensory acceptability [213]. Škara et al. (2013) [214] highlighted that the technological benefits of inulin in gluten-free breads are maximized when it is combined with other hydrocolloids. In rice-based partially baked frozen breads, the co-application of inulin with guar gum or pectin increased loaf specific volume by up to ~20%, reduced crumb hardness, and improved moisture retention during storage. These synergistic effects arise from the complementary actions of the ingredients: inulin enhances water-binding and gel formation, while hydrocolloids stabilize gas cells and mimic the viscoelastic properties of gluten [214]. In pasta products, the application of inulin in gluten-free formulations remains relatively underexplored, with only a limited number of studies currently available [215]. Gasparre and Rosell (2019) [216] evaluated the functionality of various hydrocolloids, including inulin, guar gum, xanthan gum, and CMC, in gluten-free noodles prepared with tiger nut flour. While gums such as guar and xanthan exerted the strongest structural reinforcement, inulin enhances water absorption, contributed to brighter noodle color, and yielded products with acceptable firmness and sensory quality. These findings indicate that, although its technological effect is milder compared to gums, inulin provides complementary benefits that can improve both the appearance and nutritional profile of gluten-free noodles [216]. Difonzo et al. (2022) [44] demonstrated that inulin derived from artichoke by-products, characterized by a high DP, could be successfully incorporated at levels of 5–15% into fresh gluten-free pasta. This enrichment increased dietary fiber content up to 12.4 g/100 g, lowered the predicted glycemic index (from 66.5 in the control to ~58 at 10–15%), and stimulated prebiotic activity. Sensory quality remained acceptable, with only minor effects such as slight darkening and increased compactness. At higher concentrations, however, inulin prolonged optimal cooking time, increased cooking losses due to partial leaching of soluble fibers, and reduced the swelling index, reflecting competition for water with starch [44]. In gluten-free cookies, Hajas et al. (2022) [217] investigated the incorporation of 6% (*w*/*w*) inulin in lentil-based formulations enriched with whey protein and xylitol, to evaluate its technological and sensory effects. Inulin addition led to a significant increase in cookie hardness and spread ratio, indicating structural modification of the dough. Inulin also influenced browning intensity, promoting a darker color due to enhanced Maillard reactions, especially in formulations containing whey protein. From a sensory perspective, inulin contributed to higher sweetness perception, improving flavor balance and overall acceptability. However, cookies with inulin displayed a less uniform surface appearance [217].

In summary, inulin provides complementary benefits in gluten-free systems, with promising technological and nutritional potential, but further research is required to better define its optimal applications across different matrices.

## 7. Nutritional and Health Benefits of Inulin

**Figure 5:**
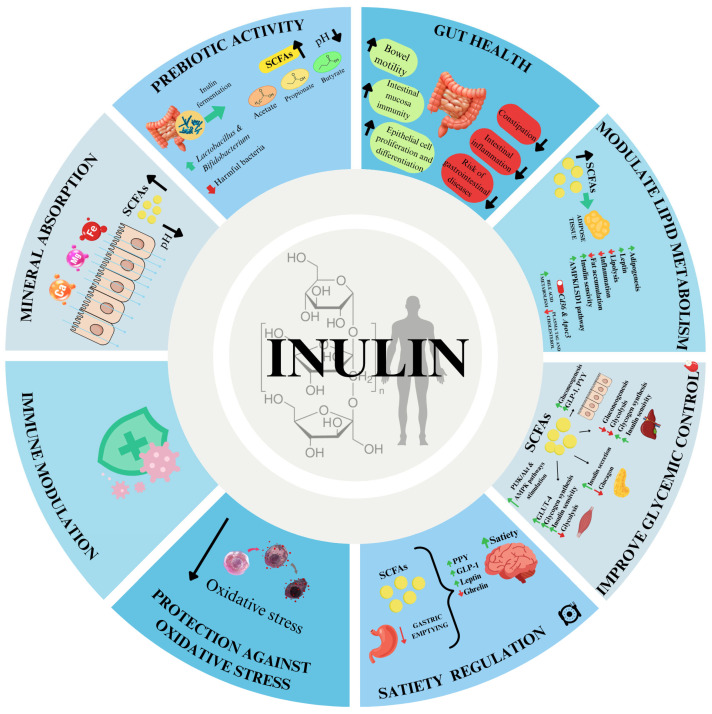
Main physiological effects of Inulin.

Inulin, beyond its role as structuring agents in food systems, is widely recognized as bioactive dietary fibers. Regular consumption of inulin has been consistently associated with a broad range of physiological benefits. These effects primarily stem from their prebiotic activity and the subsequent production of SCFAs during fermentation in the colon. In this regard, inulin represents an ingredient that bridges technological functionality with health-promoting potential. Its principal nutritional and health benefits are summarized in Figure 5 and will be discussed in the following sections.

### 7.1. Prebiotic Effect

The large intestine hosts a highly diverse microbial ecosystem, with bacterial populations broadly classified as beneficial, commensal, or potentially pathogenic. The balance between these groups is influenced by factors such as diet, stress, aging, infections, and genetic background. Microbiota dominated by *Lactobacillus* and *Bifidobacterium* are generally associated with positive health outcomes [51,64].

In this context, inulin is well established prebiotics [13,218]. To be considered a prebiotic, a compound must meet specific criteria: (i) resist gastric acidity and absorption in the upper gastrointestinal tract, (ii) be susceptible to fermentation by intestinal microbiota, and (iii) selectively stimulate the growth and/or activity of beneficial gut bacteria linked to host health and well-being [219]. The molecular structure of inulin prevents hydrolysis by human digestive enzymes, allowing them to reach the colon intact. Here, inulin acts as selective substrate for beneficial microorganisms, particularly lactobacilli and bifidobacteria [2,4,14,52,220].

Their fermentation results in the production of SCFAs—mainly acetate, propionate, and butyrate—which exert key physiological functions for the host. Acetate can be transported to peripheral organs (kidney, heart, brain, and muscle) as an energy source. Propionate, primarily metabolized in the liver, functions as a gluconeogenic precursor and contributes to the regulation of cholesterol synthesis. Butyrate is preferentially utilized by colonocytes, supporting their energy requirements, promoting cell differentiation, and exerting anti-inflammatory and anti-proliferative effects on the intestinal mucosa [2,4,26,51,106,180,220]. Collectively, SCFAs lower colonic pH, inhibiting pathogenic microorganisms’ growth while stimulating peristalsis and improving bowel regularity [106,180]. Beyond their metabolic roles, SCFAs significantly influence intestinal morphology and function by enhancing mucosal blood flow, stimulating epithelial cell proliferation and differentiation, increasing ileal motility, and supporting water and sodium absorption [2,8,52,56,180]. Evidence indicates that inter-individual variability in gut microbiota composition substantially modulates the prebiotic efficacy of inulin. Although responses to supplementation are generally consistent within the same individual, they differ markedly across subjects, being shaped by baseline SCFA profiles, habitual fiber intake, and the abundance of inulin-responsive taxa. This underscores that the prebiotic potential of inulin cannot be generalized but rather emerges from personalized host–microbiota interactions [221,222,223].

### 7.2. Reduction in Risk of Gastrointestinal Diseases

Inulin has been extensively investigated for its role in gastrointestinal health and its potential to lower the risk of intestinal disorders such as colorectal cancer (CRC), inflammatory bowel diseases (IBD), irritable bowel syndrome (IBS), and constipation [2,8,12,51,64]. A considerable body of epidemiological and experimental evidence highlights its protective effect against CRC [224,225,226,227]. Animal studies have shown that inulin supplementation reduces the formation of preneoplastic lesions, such as aberrant crypt foci, especially in chemically induced carcinogenesis models [226,228,229,230]. These protective mechanisms are linked to shifts in the intestinal microbiota—with increased populations of lactobacilli and bifidobacteria, reduced pathogenic coliforms, and lower activity of carcinogen-activating enzymes including β-glucuronidase, azoreductase, and nitroreductase [8,12,226]. FOS are rapidly fermented in the proximal colon, resulting in an early release of SCFAs, whereas long-chain inulin undergoes slower and more sustained fermentation, shifting SCFAs production toward the distal colon and thereby promoting prolonged butyrate formation [10,12,15,56,64,167,231,232]. Butyrate is crucial for colon health: it fuels colonocytes, supports epithelial differentiation, regulates cell proliferation, and promotes controlled apoptosis, thereby contributing to intestinal homeostasis [8,12,51,233,234].

Beyond cancer prevention, inulin exhibits anti-inflammatory effects relevant to IBD, including ulcerative colitis and Crohn’s disease [26,220,235,236,237]. Both preclinical and clinical studies suggest that inulin and FOS—alone or combined with probiotics—can downregulate pro-inflammatory cytokines and enhance immunomodulatory mediators such as TGF-β. Synbiotic formulations, for example, inulin combined with *Bifidobacterium longum* or selected *Lactobacillus* strains, have been shown to alleviate colitis, reduce inflammation, and improve gut barrier function [4,238,239,240,241,242].

In the context of IBS and constipation, inulin fermentation offers multiple benefits. It stimulates peristalsis, increases fecal bulk and water content, and lowers luminal pH, thereby promoting the growth of beneficial gut microbiota [26,51,56,236,243]. Constipation, a common issue particularly among older adults, can be effectively alleviated by inulin supplementation. Clinical trials have shown that even small amounts (approximately 0.74 g/day) can improve stool frequency and consistency in constipated individuals [12,244]. In 2015 the EFSA authorized a health claim under Article 13(5) of Regulation (EC) No 1924/2006. The claim acknowledges that a daily intake of 12 g/day or more of native chicory inulin contributes to normal bowel function by increasing stool frequency [245].

The gastrointestinal tolerance of inulin is strongly dose-dependent and varies considerably among individuals. While inulin and FOS are generally recognized as safe and well-tolerated, excessive consumption can lead to mild to moderate gastrointestinal symptoms, particularly in sensitive individuals. Such effects—typically including bloating, flatulence, abdominal discomfort, or diarrhea—arise from rapid fermentation of the unabsorbed fructans in the colon, leading to increased gas and osmotic activity [52,246,247]. However, the physiological response and tolerability can vary according to age, health status, and metabolic condition. In healthy adults, inulin is considered safe and well tolerated at intakes up to 40 g/day, though gastrointestinal responses may vary among individuals [247,248]. A daily intake of 10–20 g is generally recommended to obtain optimal physiological benefits, while higher amounts can be safely consumed if introduced progressively to enhance tolerance [249]. For unhealthy, overweight, or obese adults or diabetic adults should decrease the inulin intake and should not consume more than 20–25 g per day [250,251,252,253]. Patients undergoing peritoneal dialysis require additional caution, with intake preferably below 10 g/day [254]. In older adults, inulin supplementation (5–15 g/day) supports the maintenance of gut microbiota composition and intestinal function, acting as a prebiotic source of fermentable fiber. Nonetheless, excessive intake may cause gastrointestinal distress in sensitive individuals, particularly those with inflammatory bowel disease, ulcerative colitis, or food allergies [52,247]. For children, inulin supplementation is considered safe when administered at moderate levels (2–10 g/day). Individual tolerance, however, varies widely. Initial consumption may cause transient bloating or loose stools, while prolonged intake has been shown to improve stool frequency and consistency and to support healthy gut microbiota. In pediatric obesity, inulin may contribute to improved body composition and reduced fat mass [247,255]. Some studies have reported a potential interference with mineral absorption in children and adolescents, suggesting the need for balanced dietary planning [256]. During pregnancy and lactation, inulin is generally regarded as safe within moderate doses (5–10 g/day), although the evidence remains limited. Increased intakes may occasionally induce mild gastrointestinal symptoms, including flatulence or nausea, especially in women with pre-existing digestive sensitivity. Therefore, medical advice is recommended before initiating supplementation [247]. Overall, inulin demonstrates a favorable safety profile across populations when consumed within recommended limits. Careful dose management and gradual introduction are advised, particularly in individuals with gastrointestinal or metabolic conditions, to ensure optimal tolerance and benefit [247,249].

### 7.3. Enhancement of Mineral Absorption

Minerals are essential for human health, yet their absorption is often inefficient [8]. Inulin has been studied for its ability to enhance mineral bioavailability, particularly calcium (Ca), magnesium (Mg), and iron (Fe) [56,64,180]. The main mechanism involves colonic fermentation, where SCFAs lower luminal pH, increase mineral solubility, and stimulate both passive diffusion and paracellular transport, while also promoting mucosal growth and enlarging the absorptive surface [8,56,64,257]. Acidification of the lumen favors calcium ionization, improving its bioavailability and uptake, with additional contribution from ion-exchange mechanisms [64,258]. Animal studies consistently show benefits: inulin increased bone mineral content and density in rats and pigs, prevented bone loss in ovariectomized animals, and improved selenium and iron status [14,259,260,261,262,263,264]. Long chain inulin appears more effective than FOS, with approximately 20 g/kg diet identified as optimal for enhancing trace element absorption [262]. Human trials yield more variable results: in adolescent girls with low calcium absorption, an inulin–FOS mix improved Ca uptake by 18% [265], while postmenopausal women also showed better Ca and Mg absorption [266,267]. Moreover, inulin reduced hepcidin levels and improved iron absorption in children with celiac disease on a gluten-free diet, also enhancing vitamin D and E status [268,269].

### 7.4. Regulation of Food Intake and Appetite

Appetite regulation involves gastrointestinal hormones that signal the hypothalamus to balance hunger and satiety. While ghrelin stimulates food intake, anorexigenic peptides such as PYY and GLP-1 suppress appetite [64]. Inulin modulates this system through fermentation, producing SCFAs that enhance GLP-1 and PYY secretion while lowering ghrelin. These mechanisms are linked to reduced energy intake, body weight, and fat mass in animal models. Additionally, inulin slows gastric emptying, prolonging satiety and lowering overall caloric intake [26,270]. Human studies confirm these effects: FOS supplementation (21 g/day, 12 weeks) decreased body weight, fat percentage, and waist circumference in overweight adults, with reduced hunger perception [271]. Overall, inulin contributes to weight management by modulating gut hormones, delaying gastric emptying, and reducing caloric intake [8,257]. These effects, combined with its low caloric value, make inulin promising dietary strategy for obesity prevention and weight control.

### 7.5. Effect on Lipid Metabolism

Dyslipidemia, characterized by elevated triglycerides (TAG) and cholesterol levels, is a major risk factor for atherosclerosis, cardiovascular diseases, and non-alcoholic fatty liver disease [272]. With the rising prevalence of metabolic disorders linked to lifestyle and dietary habits, interest has grown in the potential lipid-lowering properties of inulin [257]. Animal studies consistently show that inulin lower plasma TAG and cholesterol, reduce hepatic lipogenesis, and protect against steatosis [273,274,275,276,277,278]. These effects are primarily attributed to SCFAs, particularly propionate, which inhibits key enzymes involved in cholesterol and triglyceride synthesis, thereby limiting hepatic lipogenesis. Moreover, SCFAs have been shown to activate the AMPK/LSD1 signaling pathway, leading to the downregulation of *Cd36* (fatty acid receptor) and *Apoc3* (lipoprotein lipase inhibitor) mRNA expression in the jejunum. This modulation improves postprandial hypertriglyceridemia and enhances bile acid metabolism [8,26,278]. High doses of FOS significantly decrease serum and liver TAG, improve the HDL/LDL ratio, and lower total cholesterol in rats [279,280]. Human trials have yielded mixed results. While some studies have shown consistency with animal experiments, others have found that inulin do not reduce TAG levels, especially in normolipidemic individuals [8,80,281,282,283]. The variability among studies likely reflects differences in baseline lipid status, dietary background, gut microbiota composition, and the type or dose of inulin used.

### 7.6. Effect on Glycemic Control and Insulin Sensitivity

The World Health Organization (WHO) defines diabetes mellitus as a chronic metabolic disorder characterized by persistently elevated blood glucose levels, which over time may damage the cardiovascular system, eyes, kidneys, nerves, and other organs. More than 90% of cases are type 2 diabetes mellitus (T2DM), associated with impaired insulin secretion, reduced tissue sensitivity to insulin, and insufficient compensatory insulin release [284,285,286]. Inulin has been investigated as dietary strategies to modulate glycemic control. Preclinical studies show that they lower hyperglycemia by reshaping gut microbiota, increasing SCFAs production—particularly propionate—enhancing insulin sensitivity, and reducing inflammation [8,249,287,288,289,290]. Human trials support these findings: Causey et al. (2000) reported that 18 g/day of inulin for 6 weeks in hypercholesterolemic men reduced fasting glucose, improved insulin sensitivity, and lowered total and LDL cholesterol [291]. Russo et al. (2010) showed that pasta enriched with 11% inulin improved fasting glucose, HbA1c, fructosamine, and HOMA-IR in healthy men, while also improving the lipid profile [292]. In women with T2DM, supplementation with 10 g/day inulin significantly decreased fasting glucose, HbA1c, insulin, and HOMA-IR, alongside reductions in inflammatory markers [293,294,295]. Mechanistically, inulin slows carbohydrate digestion and glucose absorption, while fermentation-derived SCFAs stimulate intestinal gluconeogenesis, lower hepatic glucose output, and promote GLP-1 secretion, thereby improving insulin release and β-cell function [257,296]. Moreover, SCFAs can activate AMPK and PI3K/Akt pathways, enhancing glucose uptake and utilization, further supporting the hypoglycemic effects of inulin [8,287,297].

### 7.7. Stimulation of the Immune System

Inulin exerts immunomodulatory effects primarily through modulation of gut microbiota and fermentation metabolites [8,298]. Preclinical studies show enhanced natural killer (NK) cell and macrophage activity, increased IgA production, and improved vaccine responses [299,300]. In human studies, inulin supplementation has been associated with stronger vaccine efficacy in elderly and children, reduced incidence of infections and diarrhea episodes, modulation of cytokines (↑IL-10, ↓IL-1β) [237,301,302,303,304]. Mechanistically, these effects are linked to bifidogenic activity (promoting growth of beneficial Bifidobacteria), SCFAs-mediated signaling via GPR41/43, improved intestinal barrier integrity [305]. Overall, inulin contributes to host defense by reducing inflammation and enhancing immune responses. These properties make inulin a promising dietary supplement for supporting immune function and overall health.

### 7.8. Protection Against Oxidative Stress

Oxidative stress occurs when the production of free radicals exceeds the capacity of the body’s endogenous antioxidant defenses, leading to structural damage of cell and mitochondrial membranes [12,306]. Inulin has been reported to exert antioxidant effects both in vitro and in vivo. Radical scavenging assays such as DPPH, ABTS and FRAP have demonstrated that inulin possesses measurable antioxidant capacity, although generally lower than that of vitamin C [12,307]. Animal studies corroborate these effects: inulin supplementation improved antioxidant status, and in laying hens, it was associated with enhanced oxidative defense and increased egg production rate [307]. At the cellular level, inulin extracted from *Radix* and *Rhizoma ginseng* attenuated oxidative injury in IPEC-J2 cells exposed to H_2_O_2_, reducing markers such as malondialdehyde (MDA) and lactate dehydrogenase (LDH) in a dose-dependent manner [77]. Moreover, in vitro assays demonstrated that isolated inulin exhibited notable antioxidant activity, with 75.7% DPPH radical scavenging capacity [307].

## 8. Conclusions and Future Prospective

Inulin is solidifying its role as a multifunctional ingredient in modern food systems, effectively bridging nutritional benefits with techno-functional performance. Its ability to form particle gels underlies its widespread application as a fat replacer, texture modifier, and carrier of bioactive compounds, enabling the reformulation of healthier foods without compromising sensory quality. Evidence highlights inulin’s versatility across dairy, meat, bakery, confectionery, gluten-free, and plant-based products, where it contributes to improved structure, stability, palatability, and nutritional value. Future efforts should focus on broadening the spectrum of food applications, particularly in reformulated products with reduced fat, sugar, and saturated fat content, as well as in functional, plant-based, and gluten-free formulations. Future advancements in inulin production will hinge on two key factors: refining eco-friendly extraction methods for large-scale use and maximizing the value of agricultural by-products. These combined efforts will pave the way for enhanced sustainability and seamless integration into circular economy frameworks.

In parallel, deeper investigation into non-thermal gelation approaches may enable the development of stable inulin gels suitable for thermosensitive compounds and innovative delivery systems.

## Figures and Tables

**Table 1:** Occurrence of inulin in selected plant sources.

Plant Source	Plant Part	Inulin (g/100 g FW)	References
Jerusalem artichoke (*Helianthus tuberosus*)	Tuber	17.00–20.50	[2]
Dahlia (*Dahlia pinnata*)	Tuber	15.00–20.00	[63]
Chicory (*Cichorium intybus*)	Roots	15.00–20.00	[2]
Yacon (*Smallanthus sonchifolius*)	Roots	3.00–19.00	[62]
Garlic (*Allium sativum*)	Bulb	9.00–16.00	[2]
Dendelion (*Taraxacum officinale*)	Roots	12.00–15.00	[64]
Leek (*Allium ampeloprasum*)	Bulb	3.00–10.00	[2]
Globe Artichoke (*Cynara scolymus*)	Globes	2.00–7.00	[62]
Onions (*Allium cepa*)	Bulb	3.09–4.96	[52]
Asparagus (*Asparagus officinalis*)	Roots	2.00–3.00	[52]
Wheat (*Triticum aestivum*)	Seeds	1.50–2.30	[2]
Barley (*Hordeum vulgare*)	Seeds	0.50–1.50	[52]
Banana (ripe) (*Musa acuminata*)	Fruit	0.58–1.09	[52]
Rye (*Secale cereale*)	Seeds	0.50–1.00	[52]

## Data Availability

No new data were created or analyzed in this study.

## Abbreviations

The following abbreviations are used in this manuscript:

ABTS	2,2′-Azino-bis(3-ethylBenzoThiazoline-6-Sulfonic acid)
AMPK/LSD1	AMP-activated protein kinase/lysine-specific demethylase 1 axis
CRC	ColoRectal Cancer
DM	Dry Matter
DP	Degree of Polymerization
DPPH	2,2-DiPhenyl-1-PicrylHydrazyl
EAE	Enzyme-Assisted Extraction
EFSA	European Food Safety Authority
FOS	FructoOligoSaccharideS
FRAP	Ferric Reducing Antioxidant Power
FTIR	Fourier-Transform Infrared Spectroscopy
FW	Fresh Weight
GI	Glycemic Index
GLP-1	Glucagon-Like Peptide-1
GPR41/43	G-Protein-coupled receptors 41 and 43
GRAS	Generally Recognized As Safe
HDL	High-Density Lipoprotein
HHP	High Hydrostatic Pressure
HOMA-IR	Homeostatic Model Assessment of Insulin Resistance
HWE	Hot-Water Extraction
HbA1c	Glycated Hemoglobin
IBD	Inflammatory Bowel Disease
IBS	Irritable Bowel Syndrome
IQF	Individually Quick Frozen
ITFs	Inulin-Type Fructans
JAP	Jerusalem Artichoke Powder
LDH	Lactate Dehydrogenase
LDL	Low-Density Lipoprotein
MAE	Microwave-Assisted Extraction
MDA	MalonDiAldehyde
MW	Molecular Weight
NK	Natural Killer (cells)
PEFAE	Pulsed-Electric Field Assisted Extraction
PI3K/Akt	PhosphoInositide 3-Kinase/Protein Kinase B Pathway
PYY	Peptide YY
SFE	Supercritical Fluid Extraction
SCFAs	Short-Chain Fatty Acids
TAG	TriAcylGlycerols
TGF-β	Transforming Growth Factor Beta
T_g_	Glass Transition Temperature
UAE	Ultrasound-Assisted Extraction
VGI	Volumetric Gel Index
WHO	World Health Organization
XOS	Xylooligosaccharides
XRD	X-Ray Diffraction
